# CD200R1 agonist attenuates glial activation, inflammatory reactions, and hypersensitivity immediately after its intrathecal application in a rat neuropathic pain model

**DOI:** 10.1186/s12974-016-0508-8

**Published:** 2016-02-18

**Authors:** Miriam Hernangómez, Ilona Klusáková, Marek Joukal, Ivana Hradilová-Svíženská, Carmen Guaza, Petr Dubový

**Affiliations:** Central European Institute of Technology (CEITEC), Masaryk University, Kamenice 3, 62500 Brno, Czech Republic; Department of Anatomy, Division of Neuroanatomy, Faculty of Medicine, Masaryk University, Kamenice 3, 62500 Brno, Czech Republic; Department of Functional and Systems Neurobiology, Neuroimmunology Group, Cajal Institute, Consejo Superior de Investigaciones Científicas (CSIC), Madrid, Spain

**Keywords:** Rat neuropathic pain model, Sterile nerve constriction, Neuroinflammation, Activated glial cells, Cytokines, Modulation

## Abstract

**Background:**

Interaction of CD200 with its receptor CD200R has an immunoregulatory role and attenuates various types of neuroinflammatory diseases.

**Methods:**

Immunofluorescence staining, western blot analysis, and RT-PCR were used to investigate the modulatory effects of CD200 fusion protein (CD200Fc) on activation of microglia and astrocytes as well as synthesis of pro- (TNF, IL-1β, IL-6) and anti-inflammatory (IL-4, IL-10) cytokines in the L4–L5 spinal cord segments in relation to behavioral signs of neuropathic pain after unilateral sterile chronic constriction injury (sCCI) of the sciatic nerve. Withdrawal thresholds for mechanical hypersensitivity and latencies for thermal hypersensitivity were measured in hind paws 1 day before operation; 1, 3, and 7 days after sCCI operation; and then 5 and 24 h after intrathecal application of artificial cerebrospinal fluid or CD200Fc.

**Results:**

Seven days from sCCI operation and 5 h from intrathecal application, CD200Fc reduced mechanical and thermal hypersensitivity when compared with control animals. Simultaneously, CD200Fc attenuated activation of glial cells and decreased proinflammatory and increased anti-inflammatory cytokine messenger RNA (mRNA) levels. Administration of CD200Fc also diminished elevation of CD200 and CD200R proteins as a concomitant reaction of the modulatory system to increased neuroinflammatory reactions after nerve injury. The anti-inflammatory effect of CD200Fc dropped at 24 h after intrathecal application.

**Conclusions:**

Intrathecal administration of the CD200R1 agonist CD200Fc induces very rapid suppression of neuroinflammatory reactions associated with glial activation and neuropathic pain development. This may constitute a promising and novel therapeutic approach for the treatment of neuropathic pain.

## Background

Peripheral neuropathic pain (PNP), manifesting as spontaneous pain, arises as a result of many forms of nerve damage, including traumatic nerve injury, diabetic neuropathy, HIV neuropathy, and drug-induced neuropathy [1, 2]. Among other effects, nerve injury-induced PNP is associated with inflammatory reaction and activation of glial cells in the corresponding spinal cord segments [1, 3, 4].

Experimental PNP models are predominantly based on injury to the sciatic nerve, wherein the maximum number of neuronal perikarya (98–99 %) is localized at the L4 and L5 segments [5]. Sterile chronic constriction injury (sCCI) of the sciatic nerve is a model for study of cellular and molecular changes inducing PNP after traumatic nerve injury with dominant molecular signaling from Wallerian degeneration [6]. It is well documented that hypersensitivity and ongoing pain due to peripheral nerve injury are associated with cellular and molecular changes in the dorsal horn (DH) of the spinal cord related to activation of microglial cells and astrocytes and alteration of pro- and anti-inflammatory cytokines produced by neurons, activated glia, and invaded immune cells [7–12]. There is a growing body of evidence that unilateral nerve injury results in bilateral neuroinflammatory reaction in the dorsal root ganglia and spinal cord DH [10, 13–15], thus illustrating signaling from the site of Wallerian degeneration to other compartments of the nervous system [16].

CD200 is a membrane glycoprotein of the immunoglobulin superfamily with immune suppression effect via its receptor CD200R. CD200 has an extracellular portion with two immunoglobulin domains, typical of proteins involved in cell-to-cell interaction. The CD200 receptor CD200R1 has a similar structure but with an additional intracellular domain that is susceptible to phosphorylation and involved in signal transduction [17, 18]. CD200 is highly expressed on neurons while CD200R is confined mainly to myeloid cells like macrophages and microglia [19–23]. In addition, CD200 is expressed in oligodendrocytes [23] and astrocytes [23, 24]. The interaction of CD200 with its receptor CD200R plays a significant role in maintaining microglia in a quiescent or resting state and attenuates various types of neuroinflammatory diseases [19, 25, 26]. It has been demonstrated that mice with levels of CD200 increased by spontaneous mutation in the Wld gene have less activated monocytes and increasing expression of IL-10 in the central nervous system following induction of experimental autoimmune encephalomyelitis [27]. Conversely, CD200−/− mice have been shown to display myeloid cell dysregulation, enhanced susceptibility to experimental autoimmune encephalomyelitis [28], and microglial activation [29]. Furthermore, CD200R expression can be modulated by IL-4 and IL-13 [30, 31].

Experimental studies have demonstrated that soluble CD200 fusion protein (CD200Fc), containing the ectodomain of CD200 bound to a murine IgG2a module, attenuates inflammatory diseases and reduces microglial activation [32–36]. Nevertheless, our knowledge remains limited as to the effects of CD200Fc in attenuation of glial cell activation and alteration of pro- and anti-inflammatory cytokines in PNP induced by a peripheral nerve injury. Therefore, the goal of our present study was to explore whether intrathecal application of CD200Fc might attenuate activation of glial cells in the spinal cord, modify synthesis of pro- and anti-inflammatory cytokines, and reduce behavioral signs of PNP in the sCCI model. Our results provide the first evidence that CD200Fc administration induces rapid attenuation of glial activation and proinflammatory cytokine synthesis of the spinal cord in relation to reduction of neuropathic pain signs after experimental nerve injury.

## Methods

### Animals and surgical procedures

The experiments were carried out on 120 male Wistar rats weighing 240–250 g at the beginning of experiments. The animals were housed on 12 h light/dark cycles at temperature 22–24 °C under specific pathogen-free conditions in the animal housing facility of Masaryk University. All surgical procedures were performed by one person under aseptic conditions and deep anesthesia induced by a xylazine and ketamine cocktail injected intraperitoneally (xylazine 1.6 mg/kg; ketamine 64 mg/kg). Sterilized food and water were available ad libitum. Treatment of the animals was in accordance with the European Convention for the Protection of Vertebrate Animals Used for Experimental and Other Scientific Purposes and was supervised by the institutional Ethics Committee of Masaryk University in Brno (Czech Republic).

Six naïve rats treated with CD200Fc were used to demonstrate that the CD200R agonist has no effect on basal skin sensitivity, and 18 naïve rats were utilized for comparison of glial activation by immunohistochemistry, western blot analysis, and relative cytokine messenger RNA (mRNA) levels. The left sciatic nerve was exposed at mid-thigh and three silk ligatures (Ethicon 3–0) were applied to reduce the nerve diameter by one third in the rats undergoing sCCI (*n* = 72). The left sciatic nerves of 24 sham-operated rats were only exposed without any nerve lesion. Sham- and sCCI-operated animals were left to survive for 7 days.

### Administration of CD200Fc and vehicle

At day 7 of sCCI operation, the rats were randomly divided into groups with intrathecal administration of CD200Fc for 5 h (*n* = 18; sCCI + CD200Fc5h) and 24 h (*n* = 18; sCCI + CD200Fc24h) as well as a control group with intrathecal application of artificial cerebrospinal fluid (ACSF) for 5 h (*n* = 18; sCCI + ACSF5h) and 24 h (*n* = 18; sCCI + ACSF24h). CD200Fc (Cat. No. 3355-CD; R&D Systems, Minneapolis, MN, USA) was freshly prepared in sterile ACSF [37]. The single intrathecal injection was administered by introducing a hypodermic needle into the subarachnoid space of the cisterna magna for diffusion of CD200Fc (5 μl; 2 μg/μl) or sterile ACSF (5 μl) throughout the spinal fluid over 30 s with another 30-s delay before removing the needle. To explore the effect of surgical approach on inflammatory reaction in the spinal cord, sham-operated rats were treated with ACSF (5 μl) for 5 h (*n* = 18) and 24 h (*n* = 6, only for behavioral test). A direct effect of CD200Fc on basal sensitivity was tested in six naïve rats treated with CD200Fc (5 μl; 2 μg/μl) for 5 and 24 h.

### Behavioral tests

Withdrawal thresholds for mechanical and thermal hypersensitivity were measured in ipsilateral hind paws using a dynamic plantar esthesiometer and plantar test (Ugo Basile), respectively. Rats were first acclimated in clear Plexiglas boxes for 30 min prior to testing. The paws were tested alternately with 5-min intervals between tests 1 day before operation; 1, 3, and 7 days after sCCI operation; and then 5 and 24 h after intrathecal ACSF or CD200Fc treatment. Six naïve rats were tested at 5 and 24 h after intrathecal administration to investigate a possible CD200Fc effect on basal sensitivity of animals. Five measurements were taken for each paw and test session. In the case of thermal hypersensitivity, withdrawal time was measured and the intensity radiance was set at value 50. Data for mechanical and thermal hypersensitivity were expressed as mean ± SE of withdrawal thresholds in grams and withdrawal latency in seconds, respectively. All behavioral tests were conducted in a blind manner.

### Immunohistochemical staining

The naïve rats and sCCI-operated rats treated with ACSF or CD200Fc for 5 and 24 h, as well as sham-operated and ACSF-treated rats (*n* = 6 for each group) were deeply anesthetized with a lethal dose of sodium pentobarbital (70 mg/kg body weight, i.p.) and perfused transcardially with 500 ml of heparinized (1000 units/500 ml) phosphate-buffered saline (PBS; 10 mM sodium phosphate buffer, pH 7.4, containing 0.15 M NaCl) followed by 500 ml of Zamboni’s fixative [38]. The L4–L5 spinal cord segments were removed, immersed separately in Zamboni’s fixative at 4 °C overnight, and then collected for each experimental group. The samples were washed in 20 % phosphate-buffered sucrose for 12 h and blocked in Tissue-Tek® OCT compound (Miles, Elkhart, Ind.). Serial transverse sections (12 μm) of the L4–L5 spinal cord segments were cut (Leica 1800 cryostat; Leica Microsystems, Wetzlar, Germany), collected on gelatin-coated microscopic slides, air-dried, then processed for immunohistochemical staining.

Briefly, the sections were washed with PBS containing 0.05 % Tween 20 (PBS-TW20) and 1 % bovine serum albumin for 10 min, then treated with 3 % normal donkey serum in PBS-TW20 for 30 min. The spinal cord sections were incubated with 25 μl of mouse monoclonal anti-CD11b/c antibody (OX42, 1:50; AbD Serotec, Kidlington, UK) or rabbit polyclonal anti-glial fibrillary acidic protein (GFAP, 1:250; Dako, Glostrup, Denmark) in a humid chamber at room temperature (21–23 °C) overnight or for 180 min to identify activated microglial cells and astrocytes, respectively. The immunoreaction was visualized by treatment with FITC-conjugated affinity purified donkey anti-mouse or anti-rabbit secondary antibodies (1:400; Merck Millipore) for 90 min at room temperature. A part of sections was incubated overnight with mouse monoclonal anti-CD200 antibody (OX2, 1:100; Abcam) and treated with FITC-conjugated donkey anti-mouse (1:400; Merck Millipore) secondary antibody. Spinal cord distribution of CD200R was detected by immunostaining of sections with goat polyclonal anti-CD200R (OX2R, 1:200; Santa Cruz Biotechnology, Inc. USA) antibody and biotinylated donkey anti-goat secondary antibody (1:400; Santa Cruz Biotechnology, Inc. USA). Immunoreaction was visualized by TRITC-conjugated streptavidin (1:100; Jackson Laboratories, Inc. USA). The control sections were incubated with omission of the primary antibodies (data not shown). The cell nuclei were stained using Hoechst 33342 (Sigma; St. Louis, MO, USA). Sections were mounted in Vectashield aqueous mounting medium (Vector Laboratories; Burlingame, CA, USA) and then observed and analyzed using a Leica DMLB epifluorescence microscope equipped with a Leica DFC-480 camera (Leica Microsystems GmbH, Wetzlar, Germany).

### Double immunofluorescence staining

To detect cellular localization of CD200 and CD200R proteins in the spinal cord, simultaneous immunostaining of CD200 or CD200R with corresponding cellular markers was carried out. Activated astrocytes and microglial cells were identified by immunostaining for GFAP and OX42, respectively (see above). Immunofluorescence staining with rabbit polyclonal NeuN antibody (1:500; Merck Millipore) was used to identify neurons of the spinal DH. Briefly, the spinal cord sections were immunostained for CD200 (see above), and after washing, the sections were immunolabeled with rabbit polyclonal anti-GFAP or NeuN antibody and TRITC-conjugated donkey anti-rabbit (1:400; Merck Millipore) secondary antibody. Other sections were incubated at first to visualize distribution of CD200R and then for detection of activated microglial cells using mouse monoclonal OX42 antibody and FITC-conjugated donkey anti-mouse secondary antibody (see above). The control sections of the double immunostaining were incubated as described above but with omission of CD200 or CD200R antibodies. In the controls, no immunostaining for CD200 or CD200R was detected in the spinal cord sections.

To detect CD200R in activated astrocytes, the sections immunostained for CD200R using goat polyclonal antibody and biotin-streptavidin TRITC were next immunolabeled with rabbit anti-GFAP polyclonal antibody and FITC-conjugated donkey anti-rabbit secondary antibody (see above). The control sections were incubated with the goat anti-CD200R polyclonal antibody and FITC-conjugated donkey anti-rabbit secondary antibody or with rabbit anti-GFAP polyclonal antibody, and next, a biotin-streptavidin procedure was used for visualization of CD200R. No cross reaction between the secondary and primary antibodies was detected in these control sections. Double immunostained sections were mounted in Vectashield aqueous mounting medium and analyzed using a Nikon Eclipse epifluorescence microscope equipped with a DS-Ri1 camera (NIKON, Czech Republic).

Because detection of CD200R expression in activated astrocytes is not routine, the colocalization of CD200R and GFAP immunofluorescence was analyzed by colocalization module of NIS Elements software (Nikon, Czech Republic).

### Image analysis

At least 10 sections (each separated from the next by an interval of about 80 μm) from each of the removed L4–L5 spinal cord segments for each animal per experimental group were selected for image analysis. Immunostaining area for OX42 or GFAP in spinal cord sections was measured using an NIS elements image analysis system (Laboratory Imaging Ltd, Prague, Czech Republic). Briefly, a box (4 × 10^4^ μm^2^) was placed over the lateral, central, and medial areas of DH and OX42 or GFAP immunostained structures were detected by thresholding technique after subtraction of background. The area of immunostaining for OX42 or GFAP in corresponding DH was related to the area of interest (4 × 10^4^ μm^2^) and expressed as the mean of relative area (%) ± SD.

### Western blot analysis

Rats were deeply anesthetized with a lethal dose of sodium pentobarbital (70 mg/kg body weight, i.p.). The L4–L5 spinal cord segments were detected following total laminectomy and rapidly removed, frozen in dry ice, then stored at −70 °C until elaboration for western blot or reverse transcription (RT) and real-time polymerase chain reaction (PCR).

The fresh tissue samples of L4–L5 spinal cord segments from six animals for each group were homogenized in PBS containing 0.1 % Triton X-100 and protease inhibitors (LaRoche, Switzerland) and then centrifuged at 10,000*g* for 5 min at 4 °C. Proteins were separated by SDS-polyacrylamide gel electrophoresis [39] and transferred to nitrocellulose membranes by electroblotting (Bio-Rad). Blots were blocked by 5 % milk-TBST for 1 h and incubated with mouse monoclonal OX42 antibody (1:300; AbD Serotec), rabbit polyclonal anti-GFAP (1:250; DAKO), goat polyclonal anti-CD200 (1:100; Santa Cruz Biotechnology), or anti-CD200R antibody (1:100; Santa Cruz Biotechnology) at 4 °C for 18 h. Blots were washed in PBS-TW20 and incubated with secondary antibody (goat anti-mouse or anti-rabbit, 1:1000, Immunotech; anti-goat, 1:8000, Bio-Rad), at room temperature for 1 h. Protein bands were visualized using the ECL detection kit (Amersham) on an LAS-3000 chemiluminometer reader (Bouchet Biotech) and analyzed using densitometry image software. The blots were stripped in 62.5 mM Tris–HCl, pH 6.8, containing 2 % SDS and 0.7 % β-mercaptoethanol, and they were then reprobed with a monoclonal antibody against β-tubulin (1:5000; Exbio).

### Reverse transcription and real-time polymerase chain reaction

The fresh tissue samples of L4–L5 spinal cord segments from six animals for each group were collected. Total RNA was extracted using RNeasy mini columns (Qiagen, Hilden, Germany). Contaminating genomic DNA was degraded by a treatment with DnaseI (Qiagen). The yield of RNA was determined using a Nanodrop® spectrophotometer (NanoDrop Technologies, Wilmington, DE, USA). Total RNA (1 μg in 20 μL) was reverse transcribed into complementary DNA (cDNA) using poly-dT primers and the Promega reverse transcription kit (Promega, Madrid, Spain). The oligonucleotide primer sequences used are given in Table 1. SYBR® PCR was performed using 1 μL of cDNA (corresponding to 50 ng RNA input) with 200 nM of the primers listed above (Invitrogen, Barcelona, Spain) in a Power SYBR® PCR Mastermix (Applied Biosystems, Foster City, CA, USA). Cycling conditions were as follows: 50 °C for 2 min, 95 °C for 10 min, and 40 amplification cycles of 95 °C for 15 s and 60 °C for 1 min. Samples were assayed using the Applied Biosystems PRISM 7500 sequence detection system, assaying each sample in triplicate and running a six-point standard curve in parallel. To ensure the absence of contamination with genomic DNA, a control sample using RNA as the template was run for each set of extractions. Relative quantification was obtained by calculating the ratio between the values obtained for each gene of interest and those of the 18S housekeeping gene with respect to the naïve animals.

**Table 1:** Rat primer sequences used in quantitative polymerase chain reactions

Genes	Forward	Reverse
IL-1β	5′-TTGCTTCCAAGCCCTTGACT-3′	5′-CTCCACGGGCAAGACATAGG-3′
TNF-α	5′-CATGAGCAC GGAAAGCATGA-3′	5′-CCACGAGCAGGAATGAGAAGA-3′
IL-6	5′-AAGAAAGACAAAGCCAGAGTC-3′	5′-CACAAACTGATATGCTTAGGC-3′
IL-4	5′-CCTTGCTGTCACCCTGTTCTG-3′	5′-TGCATGGAGTCCCTTTTTCTG-3′
IL-10	5′-CAGTCAGCCAGACCCACAT-3′	5′-GCTCCACTGCCTTGCTTT-3′
18S	5′-ATGCTCTTAGCTGAGTGTCCCG-3′	5′-ATTCCTAGCTGCGGTATCCAGG-3′

### Statistical analyses

Behavioral data were evaluated using Kruskal-Wallis one-way analysis with Bonferroni post hoc test and *p* values less than 0.05 were considered to be significant. To verify differences of immunostaining area, western blot and RT-PCR, a one-way ANOVA with subsequent post hoc *t* tests employing a correction of alpha according to Bonferroni for repeated measures was run with *p* < 0.05 as the level of significant differences between tested samples. Statistical differences between data of relative immunostaining area, western blot, and RT-PCR were tested by Mann-Whitney *U* test (*p* < 0.05). All statistical analyses were made using STATISTICA-12 software (StatSoft, Tulsa, OK, USA).

## Results

### CD200Fc reduces mechanical and thermal hypersensitivity of nerve-injured rats

**Fig. 1:**
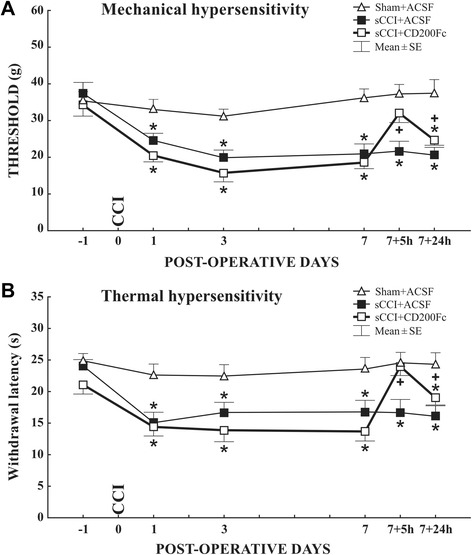
Results of behavioral tests. Mechanical (**a**) and thermal hypersensitivity (**b**) in sCCI-operated rats treated with ACSF or CD200Fc. Withdrawal thresholds for mechanical hypersensitivity and latencies for thermal hypersensitivity were significantly decreased in the ipsilateral hind paws following sCCI when compared with 1 day before surgery, and CD200Fc treatment for 5 h was able to reverse these. Both behavioral signs of PNP were nevertheless significantly weaker 24 h from CD200Fc application, although they remained still higher than 7 days after sCCI operation but before CD200Fc application. All values in **a** and **b** represent mean ± SE from six rats per group. *Asterisk* indicates statistically significant difference (*p* < 0.001) when compared with measurements 1 day before operation*; Plus sign* indicates statistical significant difference (*p* < 0.01) when compared to values of sCCI-operated animals before and after CD200Fc application. Kruskal-Wallis ANOVA followed by Mann-Whitney *U* test.

As CD200Fc appears to be beneficial when administered in cases of various diseases with an inflammatory component [34, 36, 40], we investigated whether the CD200R1 agonist has effects on PNP behavioral signs in the sCCI model. No statistically significant changes of thresholds and withdrawal latencies were measured in the hind paws of naïve rats treated with CD200Fc for 5 and 24 h, thus indicating no influence of the CD200R agonist on basal sensitivity (data not shown). Sham-operated rats displayed a small but not statistically significant mechanical and thermal hypersensitivity at days 1 and 3 with no behavioral changes after 7 days and ACSF treatment for 5 or 24 h (Fig. 1a, b). All hind paws of rats subjected to sCCI of the sciatic nerve displayed significantly decreased thresholds of mechanical (Fig. 1a) and withdrawal latencies of thermal hypersensitivity (Fig. 1b) at days 1, 3, and 7 post-operation when compared with 1 day before operation.

Rats operated on sCCI with intrathecal administration of CD200Fc for 5 h displayed significant attenuation of mechanical and thermal hypersensitivity when compared with levels before CD200Fc injection or with ACSF-treated rats. Thresholds of mechanical and withdrawal latencies of thermal hypersensitivity did drop 24 h after CD200Fc administration, but the values remained still significantly higher when compared with levels before CD200Fc treatment (Fig. 1a, b).

### CD200Fc attenuates activation of microglial cells and astrocytes in the spinal dorsal horn of nerve-injured rats

**Fig. 2:**
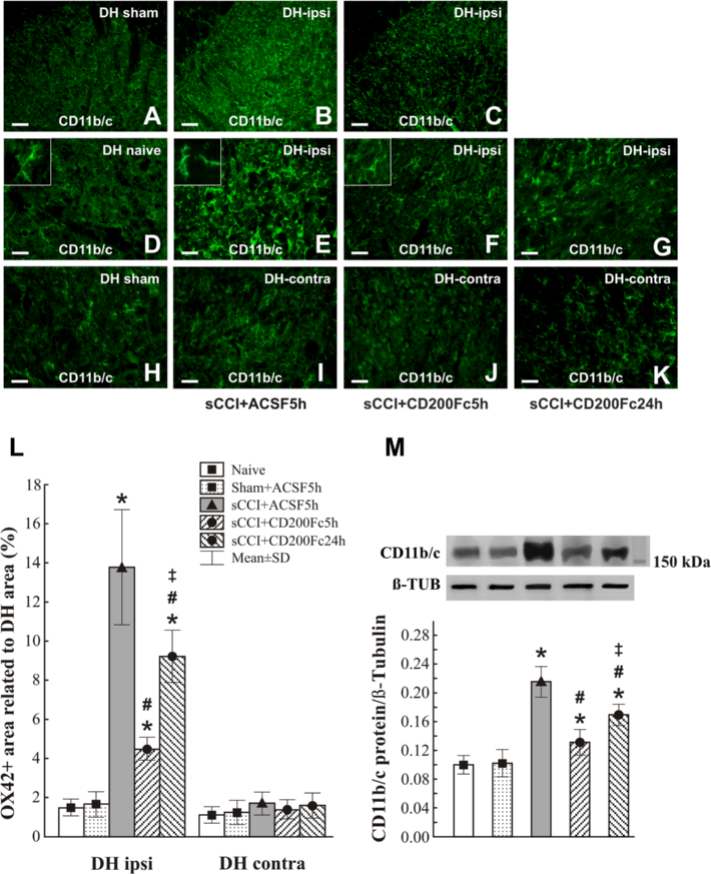
Immunofluorescence staining and quantification of CD11b/c protein. OX42 (CD11b/c protein) immunofluorescence to detect microglia in the dorsal horn (DH) of sections through L4–L5 spinal cord segments from naïve and sham-operated rats as well as sCCI-operated rats treated with ACSF or CD200Fc. Representative pictures of OX42 immunofluorescence in ipsilateral DH of the spinal cord from sham- (**a**) and CCI-operated (**b**, **c**) rats 5 h after ACSF (**a**, **b**) or CD200Fc (**c**) injection. *Scale bars* = 75 μm. A higher power magnification of OX42 immunofluorescence in DH of naïve (**d**) and sham-operated (**h**) rats and the ipsi- and contralateral DH (DH-ipsi, DH-contra) of sCCI-operated and ACSF-treated rats (**e**, **i**) as well as 5 (**f**, **j**) or 24 h (**g**, **k**) after injection with CD200Fc. *Scale bars* = 50 μm. *Insets* in **d**–**f** show typical shape of OX42-labeled microglia in DH of naïve, ACSF-, and CD200Fc-treated rats, respectively. *Scale bars* for insets = 33 μm. **l** Bar graph shows quantification of OX42 immunostaining area in DH of L4–L5 spinal cord segments from naïve and sham-operated rats as well as sCCI-operated rats for 7 days and 5 or 24 h after administration of CD200Fc or ACSF. **m** Western blot shows increased CD11b/c protein level in the spinal cord of sCCI-operated in comparison to naïve or sham-operated rats, whereas CD200Fc administration reduced this elevation. The data represent the mean ± SD optical density normalized to tubulin from six animals in each group. *p < 0.05 when compared to DH of naïve or sham-operated rats; #*p* < 0.05 when compared to DH of sCCI + ACSF rats; ++*p* < 0.05 when compared to 5-h treatment. Mann-Whitney *U* test

We investigated the regulatory effect of CD200Fc administration on microglial activation in the sCCI model of PNP because interaction of CD200 with CD200R decreases microglial activation [20, 33]. No significant difference of OX42 immunostaining was found between naïve and sham-operated rats (Fig. 2a, d, h). The OX42 immunoreactive area indicating activated microglial cells was markedly larger in ipsilateral DH of the spinal cord sections from sCCI-operated and ACSF-treated rats when compared with naïve or sham-operated animals (Fig. 2b, e). Microglial activation expressed by the OX42 immunoreactive area was significantly attenuated in ipsilateral DH sections prepared from the spinal cord of sCCI-operated rats and treated with CD200Fc for 5 h (Fig. 2c, f). However, 24 h after CD200Fc injection, the OX42 immunostaining area was enlarged, but it remained smaller than in control group of sCCI-operated and ACSF-treated rats (Fig. 2g). In contrast to ipsilateral DH, no significant extension of OX42 immunoreactive area was detected in the contralateral DH of sCCI-operated rats treated with ACSF or CD200Fc in comparison to naïve or sham-operated rats (Fig. 2i–k). The microglial cells immunostained for OX42 displayed the typical activated, amoeboid morphology in sections of L4–L5 spinal cord segments from rats sCCI-operated and treated with ACSF, whereas those in sections from CD200Fc-treated rats exhibited a ramified morphology similar to microglia of the naïve animals (insets in Fig. 2d–f). Quantitative changes of microglial activation detected by OX42 immunostaining area in the spinal cord sections of naïve, sham- and sCCI-operated, and ACSF- or CD200Fc-treated rats (Fig. 2l) were confirmed by western blot analysis of CD11b/c protein (Fig. 2m).

**Fig. 3:**
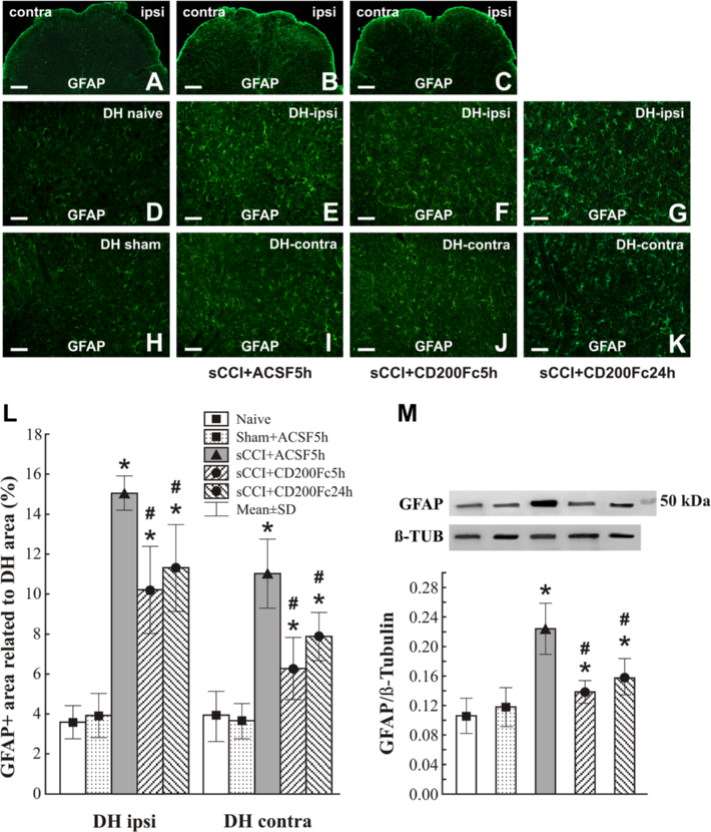
Immunofluorescence staining and quantification of GFAP protein. GFAP-immunoreactive astrocytes in the dorsal horn (DH) of sections through L4–L5 spinal cord segments from naïve rats and sham-operated rats as well as sCCI-operated rats treated with ACSF or CD200Fc. Representative pictures of GFAP immunostaining in ipsilateral (ipsi) and contralateral (contra) DH of the spinal cord from sham- (**a**) and CCI-operated (**b**, **c**) rats 5 h after ACSF (**a**, **b**) or CD200Fc (**c**) injection. *Scale bars* = 100 μm. A higher power magnification of GFAP immunostaining in DH of naïve (**d**) and sham-operated (**h**) rats and the ipsi- and contralateral DH (DH-ipsi, DH-contra) of sCCI-operated and ACSF-treated rats (**e**, **i**) as well as rats 5 (**f**, **j**) or 24 h (**g**, **k**) after injection with CD200Fc. *Scale bars* = 50 μm. **l** Bar graph shows quantification of GFAP-immunoreactive area in DH of L4–L5 spinal cord segments from naïve and sham-operated rats as well as sCCI-operated rats for 7 days and 5 or 24 h after administration of CD200Fc or ACSF. **m** Western blot confirmed the increase of GFAP protein level in the spinal cord of sCCI-operated in comparison to naïve or sham-operated rats. The GFAP elevation was reduced by CD200Fc administration. The data represent the mean ± SE optical density normalized to tubulin from six animals in each group. *p < 0.05 when compared to DH of naïve or sham-operated rats; #*p* < 0.05 when compared to DH of sCCI + ACSF rats. Mann-Whitney *U* test

As CD200Fc appears to attenuate microglial activation in the sCCI-operated rats, we investigated whether intrathecal application of CD200Fc would also affect astroglial activation. Lumbar spinal cord sections prepared from rats 7 days after sCCI operation and treatment with ACSF displayed a bilateral enlargement of GFAP immunostaining area in DH when compared to the spinal cord sections of naïve or sham-operated rats (Fig. 3a–b, d–e, h–i). In comparison to ACSF treatment, intrathecal administration of CD200Fc bilaterally reduced the extent of astroglial GFAP immunostaining in L4–L5 spinal cord sections from sCCI-operated rats (Fig. 3c, f, j). The GFAP-immunostained area of activated astrocytes was enlarged at 24 h after CD200Fc treatment, but it still remained lower than in the spinal cord of sCCI-operated and ACSF-treated rats. The quantitative changes of GFAP-immunoreactive areas (Fig. 3l) indicating activation state of astrocytes were confirmed by western blot analysis of GFAP protein in the spinal cord of naïve, sham-operated, and sCCI-operated and treated rats (Fig. 3m).

### Cellular distribution and regulation of CD200 and CD200R1 levels in the spinal cord of nerve-injured and CD200Fc-treated rats

**Fig. 4:**
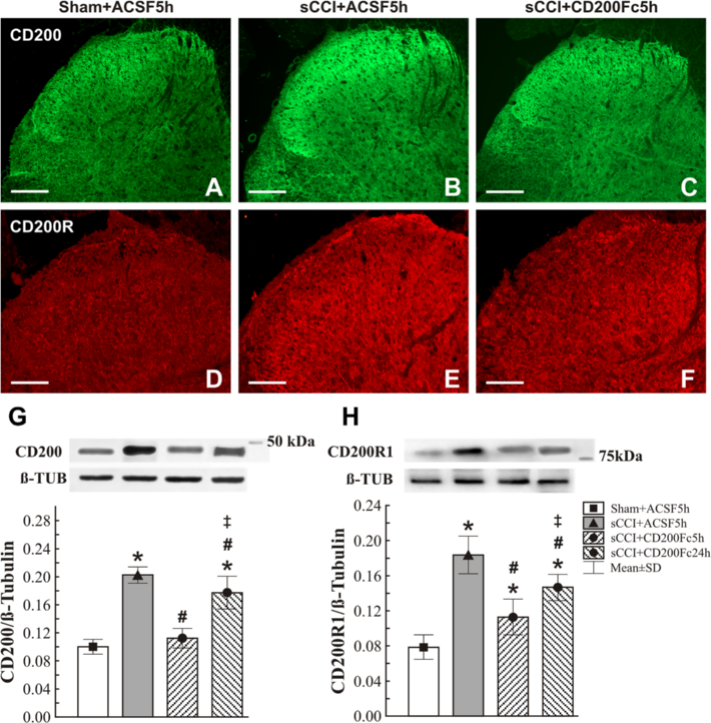
Effect of CD200Fc on CD200 and CD200R1 protein levels. Effect of CD200Fc on CD200 and CD200R1 protein levels of the L4–L5 spinal cord segments from sCCI-operated rats. sCCI of the sciatic nerve and ACSF treatment increased intensity of CD200 and CD200R immunofluorescences in DH of spinal cord sections when compared with those from sham-operated rats. Intrathecal CD200Fc administration significantly attenuated elevation of CD200 and CD200R immunofluorescences in the spinal cord sections of sCCI-operated rats (**a**–**f**). *Scale bars* = 100 μm. The CD200 and CD200R immunofluorescence changes were confirmed by western blot analysis of CD200 and CD200R proteins (**g**, **h**). *Upper panels* of **g** and **h** illustrate representative western blotting bands for CD200 and CD200R in the L4–L5 spinal cord segments from sham-operated (Sham), sCCI-operated and ACSF-treated (sCCI + ACSF5h) rats, as well as rats operated for sCCI and 5 (sCCI + CD200Fc5h) or 24 h (sCCI + CD200Fc24h) after administration of CD200Fc. *Lower panels* show mean density ± SE of individual CD200 and CD200R1 protein bands in triplicate analysis of six animals after normalization to tubulin. *p < 0.05 when compared to naïve rats; #*p* < 0.05 when compared to sCCI + ACSF rats; ++*p* < 0.05 when compared to 5 h after administration of CD200Fc. Mann-Whitney *U* test

To determine precisely whether exogenous soluble CD200Fc might modify membrane-bound CD200 and CD200R1 expression in the spinal cord after sCCI operation, we analyzed their proteins by immunofluorescence staining and western blotting in naïve and sham-operated rats in comparison with those of sCCI-operated animals after ACSF and CD200Fc administration for 5 h. The CD200 immunostaining was dominantly observed in DH with a higher intensity in the spinal cord sections from sCCI-operated and ACSF-treated rats when compared with that from sham-operated or sCCI-operated and CD200Fc-treated rats (Fig. 4a–c). Similarly, a distinctly increased immunostaining for CD200R1 was found in DH of spinal cord sections of sCCI-operated and ACSF-treated rats when compared with sections of the spinal cord from sham-operated rats (Fig. 4d–f). The increased CD200 and CD200R1 proteins in the spinal cord of sCCI-operated and ACSF-treated as well as the protein decrease after CD200Fc injection were confirmed by western blot analysis (Fig. 4g, h).

**Fig. 5:**
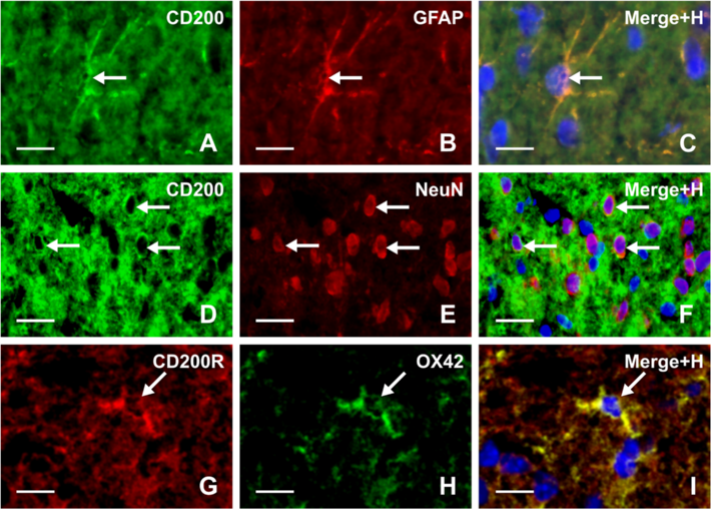
Double immunostaining to detect CD200 protein in astrocytes and neurons as well as CD200R in microglia. Double immunostaining of sections through DH of L4–L5 spinal cord segments from sCCI-operated and ACSF-treated rats. Immunofluorescence for CD200 (**a**) and GFAP (**b**) are colocalized (**c**), evidencing that CD200 protein is expressed by astrocytes (*arrows*). In addition, double immunostaining for CD200 (**d**) and NeuN (**e**) detected a presence of CD200 protein also in neurons (**f**, *arrows*). Immunolabeling for CD200R (**g**) and OX42 (**h**) was colocalized, illustrating that activated microglial cells display CD200R protein (**i**, *arrow*). Merged figures (**c**, **f**, **i**) contain also blue channel of Hoechst stained nuclei. *Scale bars* = 30 μm

**Fig. 6:**
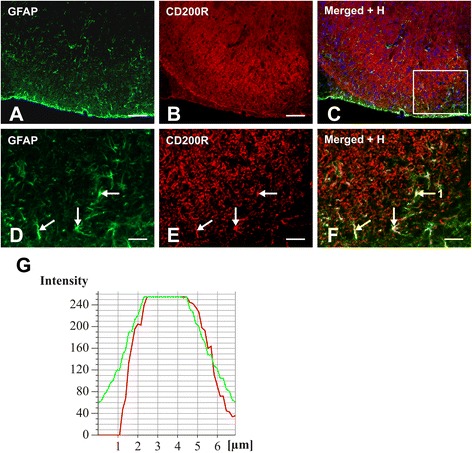
Double immunostaining to detect CD200R protein in astrocytes. Double immunostaining of section through DH of L4–L5 spinal cord segments from sCCI-operated and ACSF-treated rats to detect CD200R protein in GFAP positive astrocytes. Immunofluorescence staining for GFAP (**a**, **d**) and CD200R (**b**, **e**) was colocalized as shown in merged figure (**c**, **f**). *Scale bars* = 100 μm. **d**–**f** illustrate a higher power magnification of area limited by a box in **c**. Evidence of CD200R in activated astrocytes was confirmed by image analysis of three cells in the section using NIS Elements software. Mander’s overlap was 0.965265, 0.929336, and 0.960253 for cells indicated by *arrows. Scale bars* = 30 μm. **g** illustrates representative profile intensity of *red* (CD200R) and *green* (GFAP) channels in cell tagged 1

Double immunostaining of spinal cord sections of sCCI-operated and ACSF-treated rats revealed CD200 immunoreaction in both NeuN+ neurons and GFAP+ astrocytes (Fig. 5a–f). The CD200R immunofluorescence was detected in neuropil of the spinal cord sections and double immunostaining displayed colocalization of CD200R and OX42 to illustrate presence of CD200R in activated microglial cells (Fig. 5g–i). When GFAP immunostaining corresponding with astrocyte activation was reduced after CD200Fc treatment, we sought to observe whether activated astrocytes could express CD200R1. Surprisingly, CD200R immunolabeling was colocalized with GFAP immunostaining, indicating expression of the receptor in activated astrocytes of the spinal cord from sCCI-operated rats (Fig. 6a–f). The unexpected colocalization was verified by image analysis and measurement of indexes using NIS Elements. Mander’s overlap measured in double immunostained cells indicated by arrows (Fig. 6d–f) was 0.965265, 0.929336, and 0.960253. In addition, profile intensity of red (CD200R) and green (GFAP) channels in the cells also indicated colocalization of CD200R and GFAP immunostaining (Fig. 6g).

### Intrathecal CD200Fc administration reduces proinflammatory and enhances anti- inflammatory cytokine mRNAs in lumbar spinal cord segments of nerve-injured rats

To determine the immediate effect of CD200Fc treatment on cytokine synthesis in the spinal cord 7 days after sCCI operation, RT-PCR was carried out using primers specific to pro- (TNF, IL-1β, IL-6) and anti-inflammatory (IL-4, IL-10) cytokines.

**Fig. 7:**
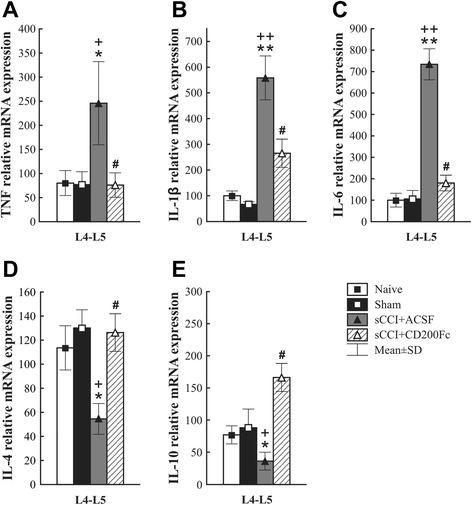
RT-PCR analysis of cytokine RNAs in the spinal cord after CD200Fc administration. Effect of CD200Fc on TNF, IL-1β, IL-6, IL-4, and IL-10 mRNAs expression in L4–L5 segments of spinal cord from sCCI-operated rats for 7 days. sCCI of the sciatic nerve and ACSF treatment increased levels of TNF (**a**), IL-1β (**b**), and IL-6 (**c**) as well as decreased IL-4 (**d**) and IL-10 (**e**) mRNA expression in the lumbar spinal cord segments. Intrathecal CD200Fc administration prevented the increase of TNF, IL-1β, and IL-6 as well as the decrease of IL-4 and IL-10 mRNAs expression in the lumbar spinal cord segments. The mRNA expression of each gene (*n* = 6 per group) was normalized to that of the 18S gene and the data represent the mean ± SD. *p < 0.05, **p < 0.001 when compared to naïve rats; +*p* < 0.05, ++*p* < 0.001 when compared to sham-operated rats; #*p* < 0.05 when compared to sCCI + ACSF rats. Mann-Whitney *U* test

As expected, L4–L5 segments of the spinal cord from sCCI-operated and ACSF-treated rats displayed robust elevation of relative mRNAs of proinflammatory (TNF, IL-1β, IL-6) and a decrease of anti-inflammatory cytokines (IL-4, IL-10) when compared with spinal cord segments of naïve or sham-operated rats. After 5 h, intrathecal administration of CD200Fc produced normalization of TNF mRNA expression (Fig. 7a) and significant reduction in relative expression of IL-1β and IL-6 mRNAs (Fig. 7b–c) in comparison with ACSF-treated rats. Conversely, relative levels of IL-4 and IL-10 mRNAs were simultaneously increased close to (in the case of IL-4) or exceeding (for IL-10) those levels for spinal cord segments from naïve or sham-operated rats (Fig. 7d–e).

## Discussion

Many experimental models of PNP are principally based on peripheral nerve injury providing a partial disconnection of axons from their neuronal bodies [6, 41]. The original model of CCI using four ligatures of chromic gut (4–0) loosely tied around the sciatic nerve [42] is not suitable for distinguishing neuroinflammatory reaction induced by a thread material [43] and/or Wallerian degeneration of injured axons [6]. Therefore, sCCI of the sciatic nerve in our experiments was prepared using 3–0 sterilized thread (Ethicon) under aseptic conditions to study the effect of CD200Fc on spinal glial activation and cytokine reactions in consequent Wallerian degeneration induced by traumatic nerve compression. It is well known that PNP induced by nerve injury is related to activation of glial cells and upregulation of proinflammatory cytokines in the spinal cord, and their reduction has been associated with alleviation of behavioral signs [44–47].

CD200/CD200R is a regulatory system of inflammation that plays a relevant role in various diseases when CD200 ligand binding with its receptor is impaired [22, 25, 29]. In contrast, enhanced signaling of CD200R by CD200Fc treatment alleviates pathological effects of inflammation [32, 34–36]. However, the anti-neuroinflammatory effects of CD200Fc in neuropathic pain have not heretofore been investigated. The present study provides the first evidence that CD200Fc attenuates activation of glial cells and proinflammatory cytokines, as well as mechanical and thermal hypersensitivity in an experimental model of PNP immediately after its intrathecal administration.

CD200Fc has been applied intraperitoneally [35], subcutaneously [34], or intracerebroventricularly [32] in various experimental models of inflammatory diseases. As CD200Fc has a half life of just a few hours [48], we applied the CD200R soluble ligand intrathecally to achieve direct and rapid diffusion of CD200Fc into DH of the rat spinal cord. We cannot exclude the possibility that a small portion of the drug administered into the cisterna magna will retrogradely reach the ventricular system and surrounding structures playing a role in the effects described here. However, decreased glial activation and modulation of cytokine mRNAs and CD200/CD200R changes revealed that CD200Fc diffused into the lumbar level of spinal cord.

### CD200Fc attenuates glial activation of the spinal cord and neuropathic pain behavioral signs

Activation of microglial cells and astrocytes after nerve injury is strongly associated with induction and maintenance of mechanical and thermal hypersensitivity, and depression of the glial activities alleviates the behavioral signs of PNP [46, 49–51]. Microglial cells are accumulated in the spinal cord at the ipsilateral side to nerve injury and change their morphology from a “resting state” in which the cell bodies are small with long and thin processes into an “activated state” in which the cells present an amoeboid shape with their bodies enlarged and fine processes retracted [52, 53]. Microglial activation after peripheral nerve injury is generally detected by immunocytochemical staining using the OX42 antibody, directed against a complement receptor 3 antigen (CD11b/c) or antibody against ionized calcium binding adapter molecule 1 (Iba1) [54, 55]. However, there is convincing evidence that enlarged OX42 immunostaining area measured by image analysis is related with microglial activation after nerve injury [53].

Ipsilateral DH of the spinal cord from sCCI + ACSF in contrast to naïve or sham-operated rats displayed enlarged areas of OX42 immunofluorescence and appearance of OX42+ microglial cells with the shapes indicating their activated state. The OX42 immunoreaction area was significantly reduced in DH of sCCI-operated and CD200Fc-treated rats, thus showing very rapid effect on the marker of activated microglia. The changes of microglial cell activation induced by CD200Fc were confirmed by western blot analysis of the CD11b/c protein. Our results are in agreement with other experimental studies in that CD200Fc rapidly reduced the markers of microglial activation [32, 34, 56].

Astrocytes are more dominant than microglia in the spinal cord, and they have been investigated predominantly in relation to their supportive functions for neurons [57]. Moreover, activated astrocytes are a major source of cytokines and may contribute significantly to induction and maintenance of PNP [45, 49]. Astrocytes respond to various physiological or pathological stimuli with the increased expression of GFAP that is generally considered to be a marker for astrocyte activation [58]. It has been experimentally demonstrated that GFAP upregulation in the spinal cord correlates well with nerve injury-induced PNP [49] and that suppression of astrocyte activation by fluorocitrate alleviates PNP symptoms [59]. In our experiments, as demonstrated by GFAP immunofluorescence areas and western blot analysis of GFAP protein, astrocytes became activated in sCCI-operated and ACSF-treated rats and were reduced bilaterally in DH of the spinal cord after treatment with CD200Fc. This suppression of activated astrocytes was much less than microglial cells which is in accordance with treatment by selective inhibitors of glial activation [55]. Our results of western blot analysis were obtained from whole L4–L5 segments containing possible changes also in the ventral horn of the spinal cord. However, OX42 and GFAP protein levels correlated with quantitative alterations of corresponding immunofluorescence were measured only in DH of the spinal cord.

Our present results evidence for the first time that intrathecal application of CD200Fc for 5 h diminished activation of both microglia and astrocytes in DH of the spinal cord after sciatic nerve injury. This rapid but reversible suppression of microglia and astrocyte activations runs in parallel with alleviation of both mechanical and thermal hypersensitivity. Because both activated microglial cells and astrocytes displayed CD200R, its activation by CD200Fc explains attenuation of the glial activation and consequent alleviation of nerve injury-induced PNP like in experimental studies using other modulators of glial activation [34, 46, 60]. Very rapid suppression of activated glial cells by CD200Fc was not surprising inasmuch as intrathecal injection of other drugs has been shown to abolish glial activation within hours [46, 61]. The CD200Fc effect was already weakened 24 h from single injection, however, thus indicating the necessity of repeated administration for longer PNP attenuation, as is usual for other molecules modulating neuroinflammation or pain [62, 63].

### Expression of CD200 and CD200R in the dorsal horn of spinal cord

CD200 protein is constitutively expressed in neurons and to a lesser extent in reactive astrocytes while CD200R is expressed by microglia [21, 23]. The changes of CD200 and CD200R1 protein levels in the spinal cord after nerve injury and following administration of CD200Fc have not previously been described. The CD200 and CD200R immunostaining was predominantly found in the spinal dorsal horn and both immunofluorescence staining and western blot analysis revealed significantly higher levels of CD200 and CD200R proteins in the spinal cord after sCCI of the sciatic nerve. In contrast to neurodegenerative diseases [19, 25, 64], sciatic nerve injury probably activated endogenous regulatory mechanisms including upregulation of CD200/CD200R. Intrathecal CD200Fc treatment following sciatic nerve injury suppressed CD200 and CD200R proteins in the spinal cord to levels close to those of naïve or sham-operated rats. The rapid regulation of the CD200 and CD200R proteins was parallel with activation or deactivation of microglia cells and astrocytes detected by CD11b/c or GFAP markers, respectively.

Using double immunostaining, we confirmed a presence of CD200 protein in neurons and astrocytes as well as CD200R in microglial cells as described earlier [21, 23]. However, there are no uncompromising results about expression of CD200R in astrocytes. We detected colocalization of GFAP and CD200R immunostaining in the spinal DH of sCCI-operated and ACSF-treated rats, thus indicating a presence of CD200R protein also in astrocytes activated by nerve injury. Activation of both microglia and astrocytes might therefore be directly modulated by CD200Fc through stimulation of CD200R. Intense cross talk between glial cells in the spinal DH is induced in the initiation phase of injury-induced PNP [8, 55, 65]. CD200/CD200R-mediated suppression of neuroinflammation may occur not only via neuron-microglia interactions but also via glia-glia interactions [23]. In addition to neurons, increased expression of CD200 in reactive astrocytes may contribute to control of microglial activation, while CD200R upregulation probably allows a modulation of astrocyte activation by contact with CD200+ neurons.

### CD200Fc decreases proinflammatory and increases anti-inflammatory cytokine mRNAs in the spinal cord of nerve-injured rats

In the present time, there is no evidence about direct operation of CD200/CD200R dysbalance in neuropathic pain induction. It is generally accepted that CD200/CD200R modulatory system is involved in suppression of inflammation and glial activation. Although the recruitment of neutrophils, macrophages, and lymphocytes from blood might participate in the neuroimmune response of the spinal cord to nerve injury, the activation of microglia and astrocytes undoubtedly plays a pivotal role in production of cytokines and chemokines [10, 12, 54].

A nerve injury induces elevation of proinflammatory cytokine levels and their synthesis (mRNA) in the spinal cord associated with development and maintenance of PNP [66–68]. Experimental studies have demonstrated that suppression of proinflammatory cytokines like TNF, IL-1β, and IL-6 is necessary for efficacious attenuation of mechanical and thermal hypersensitivity [67, 69, 70]. In contrast, increased synthesis or delivery of IL-10 has been shown to attenuate proinflammatory cytokine levels and alleviated PNP [71]. Similarly, other anti-inflammatory cytokines, such as IL-4, also have been decreased in the CCI model of PNP and IL-4 deficiency has been associated with mechanical hypersensitivity [72]. Accumulating evidence indicates that nerve injury induces upregulation of pro- and anti-inflammatory cytokines in activated microglia and astrocytes in relation with development and maintenance of PNP [7, 10, 44]. Our results from RT-PCR showed increase of proinflammatory cytokine (TNF, IL-1β, IL-6) and decrease of anti-inflammatory cytokine (IL-4, IL-10) mRNAs in the spinal cord of nerve-injured rats in comparison with naïve and sham-operated animals. Intrathecal CD200Fc treatment diminished elevation of proinflammatory cytokine (TNF, IL-1β, IL-6) mRNAs and simultaneously ameliorated deficit of IL-4 and IL-10 mRNAs induced by unilateral sCCI of the sciatic nerve. This modulation of pro- and anti-inflammatory cytokine mRNAs by CD200Fc treatment was simultaneous with glia deactivation and attenuation of mechanical and thermal hypersensitivity.

CD200R1 stimulation suggests that CD200/CD200R1 interaction is involved in suppression of the proinflammatory phenotype of microglial cells [32–34, 73] and astrocytes. In addition, an in vitro study showed how microglia incubation with CD200-bearing astrocytic membrane preparations mitigates the LPS-induced increase in mRNA expression of the proinflammatory cytokines TNF, IL-1β, and IL-6 [74]. In line with our findings, CD200Fc treatment significantly decreased the production of TNF and IL-6 but not of IL-10 or TGF-β in microglial cells of mice with experimental autoimmune encephalomyelitis [34]. Referring to anti-inflammatory cytokines, it has been pointed out that there is a possible involvement of CD200Fc in increasing IL-4 and IL-10 in microglia cells [33, 56]. Moreover, an increased expression of IL-10 in relation to enhanced neuronal levels of CD200 has been also found in the central nervous system of experimental autoimmune encephalomyelitis mice [27].

The present results indicate that sciatic nerve injury-induced upregulation of proinflammatory cytokines in parallel with elevation of the CD200/CD200R regulatory system. An enhanced signaling of CD200R1 by CD200Fc treatment attenuated activation of microglial cells and astrocytes simultaneously with a decrease of proinflammatory and increase of anti-inflammatory cytokine synthesis. Due to reduction of the inflammatory reaction by CD200Fc, expression of the CD200/CD200R regulatory system was diminished.

In conclusion, our results suggest that support of the CD200/CD200R regulatory system by administration of the CD200R1 agonist CD200Fc induces very rapid suppression of neuroinflammatory reactions associated with glial activation and PNP development. This may constitute a promising and novel therapeutic approach for the treatment of PNP. Additional experiments with nerve injury models are nevertheless needed, and particularly with repeated CD200Fc treatment to prolong its effect.

## Conclusions

The present results indicate that sCCI of the sciatic nerve induced upregulation of proinflammatory cytokines in parallel with elevation of the CD200/CD200R regulatory system. An enhanced signaling of CD200R1 by administration of the CD200R1 agonist CD200Fc induces very rapid suppression of glial activation in the spinal cord simultaneously with a decrease of proinflammatory and increase of anti-inflammatory cytokine synthesis associated with attenuation of PNP development. Due to reduction of the inflammatory reaction by CD200Fc, expression of the CD200/CD200R regulatory system was diminished.

Support of the CD200/CD200R regulatory system by administration of the CD200R1 agonist CD200Fc may constitute a promising and novel therapeutic approach for the treatment of PNP. Additional experiments with nerve injury models are nevertheless needed and particularly with repeated CD200Fc treatment to prolong its effect.
